# Clonal Analysis in Recurrent Astrocytic, Oligoastrocytic and Oligodendroglial Tumors Implicates *IDH1*- Mutation as Common Tumor Initiating Event

**DOI:** 10.1371/journal.pone.0041298

**Published:** 2012-07-23

**Authors:** Ulrike Lass, Astrid Nümann, Kajetan von Eckardstein, Jürgen Kiwit, Florian Stockhammer, Jörn A. Horaczek, Julian Veelken, Christel Herold-Mende, Judith Jeuken, Andreas von Deimling, Wolf Mueller

**Affiliations:** 1 Department of Neuropathology, Institute of Pathology, University Hospital Heidelberg, Heidelberg, Germany; 2 Clinical Cooperation Unit Neuropathology, German Cancer Center (DKFZ), Heidelberg, Germany; 3 Department of Neurology, Universitätsmedizin Charité, Berlin, Germany; 4 Department of Neurosurgery, HELIOS Klinikum Berlin Buch, Berlin, Germany; 5 Department of Neurosurgery, University Hospital Göttingen, Göttingen, Germany; 6 Department of Neurosurgery, Vivantes Klinikum Neukölln, Berlin, Germany; 7 Department of Neurosurgery, University Hospital Heidelberg, Heidelberg, Germany; 8 Department of Pathology, Nijmegen Center for Molecular Life Sciences, Radboud University Nijmegen Medical Centre, Nijmegen, The Netherlands; The University of Chicago, United States of America

## Abstract

**Background:**

To investigate the dynamics of inter- and intratumoral molecular alterations during tumor progression in recurrent gliomas.

**Methodology/Principal Findings:**

To address intertumoral heterogeneity we investigated non- microdissected tumor tissue of 106 gliomas representing 51 recurrent tumors. To address intratumoral heterogeneity a set of 16 gliomas representing 7 tumor pairs with at least one recurrence, and 4 single mixed gliomas were investigated by microdissection of distinct oligodendroglial and astrocytic tumor components. All tumors and tumor components were analyzed for allelic loss of 1p/19q (LOH 1p/19q), for *TP53-* mutations and for R132 mutations in the *IDH1* gene. The investigation of non- microdissected tumor tissue revealed clonality in 75% (38/51). Aberrant molecular alterations upon recurrence were detected in 25% (13/51). 64% (9/14) of these were novel and associated with tumor progression. Loss of previously detected alterations was observed in 36% (5/14). One tumor pair (1/14; 7%) was significant for both. Intratumoral clonality was detected in 57% (4/7) of the microdissected tumor pairs and in 75% (3/4) of single microdissected tumors. 43% (3/7) of tumor pairs and one single tumor (25%) revealed intratumoral heterogeneity. While intratumoral heterogeneity affected both the *TP53*- mutational status and the LOH1p/19q status, all tumors with intratumoral heterogeneity shared the R132 *IDH1-* mutation as a common feature in both their microdissected components.

**Conclusions/Significance:**

The majority of recurrent gliomas are of monoclonal origin. However, the detection of divertive tumor cell clones in morphological distinct tumor components sharing *IDH1*- mutations as early event may provide insight into the tumorigenesis of true mixed gliomas.

## Introduction

Presently, glioma therapy experiences a gradual paradigm shift replacing former “one-fit-all” strategies towards the development of more individual, patient- and tumor- tailored therapeutic approaches.

Astrocytomas, oligodendrogliomas and oligoastrocytomas account for the majority of glial tumors. In typical cases astrocytomas and oligodendrogliomas reveal distinct morphological features that help establish the diagnosis. While astrocytomas frequently feature fibrillary neoplastic astrocytes, oligodendrogliomas are composed of monomorphic cells with uniform round nuclei and perinuclear halos on paraffin sections (“honeycomb” appearance). Immunohistochemical detection of glial fibrillary acidic protein (GFAP) additionally helps identify astrocytic neoplasms. Oligoastrocytomas are defined as diffusely infiltrating gliomas composed of a conspicuous mixture of two distinct cell types morphologically resembling the tumor cells in oligodendroglioma and diffuse astrocytoma.

Recent identifications of distinct molecular features in both oligodendroglial and astrocytic tumors that predict response rate to defined therapeutic strategies and thus directly impact the clinical outcome of individual patients paved the way for this development. While in numerous studies combined LOH1p/19q proofed to be the decisive alteration predicting better tumor prognosis and therapy response in oligodendroglial tumors [1], [2], [3], [4], there is evidence that the methylation status of the *MGMT-* promoter may be of similar importance for the therapy and long term prognosis of malignant astrocytic tumors [5], [6], [7]. Therapy that aims at defined molecular alterations necessitates anteceding molecular testing. For the majority of patients, however, molecular testing is confined to the initial tumor, only. Upon tumor recurrence clinicians often refrain from additional cytoreductive surgical interventions and base their therapeutic decisions on morphological, immunohistochemical and molecular features defined by the initially removed tumor tissue. This approach postulates that molecular features persist during tumor recurrence. It ignores the possibility that the tumor may develop novel therapy- decisive molecular alterations during tumor progression. It also neglects the possibility that therapy itself may influence the molecular properties of recurrent tumors. Prominent example of therapy- induced molecular alterations in gliomas which themselves ensue therapeutic consequences are *MSH6* mutations in glioblastomas which arise during temozolomide chemotherapy and which are able to convey temozolomide resistance in affected tumors [8], [9], [10]. To address the issue of tumor clonality during tumor progression we investigated non-microdissected tumor tissue of a total of 106 gliomas representing a total of 51 recurrent tumors. As indicators of clonality we selected three molecular markers that are affected at high frequency in either predominantly astrocytic tumors, *i.e. TP53*- mutation, or in predominantly oligodendroglial tumors [11], [12], *i.e.* LOH1p/19q, or show similar frequencies in both, i.e. *IDH1*- mutations affecting codon 132. To elucidate clonal diversity within single and recurrent gliomas we investigated oligodendroglial and astrocytic tumor components of a total 16 tumors representing 7 tumor pairs with at least on recurrence and 4 single tumors by microdissection. While oligodendroglial and astrocytic tumor components were readily identifiable in the oligoastrocytomas, small but distinct tumor components reminiscent of astrocytoma or oligodendroglioma enabled microdissection in the oligodendroglial tumors and one GBM, respectively. All tumor components were also analyzed for *TP53*- mutations, LOH1p/19q and R132 *IDH1*- mutations. As combined LOH on 1p/19q is frequently observed in pure oligodendrogliomas, potentially driven by translocation events [11], [12] and pure astrocytomas are more prone to harbor *TP53* mutations, these alterations allow for the molecular distinction of tumor components of either more astrocytic or oligodendroglial lineage. What is more LOH 1p/19q is not commonly found in pure astrocytomas and *TP53* mutations are infrequent in oligodendrogliomas. Mixed gliomas can harbor both LOH1p/19q and *TP53-* mutations at lower rates as their respective pure counterparts. The current study elucidates the possibility of the existence of bi- or multiclonal tumors by microdissection. It investigates molecular alterations during tumor recurrence also taking into account possible effects of therapeutic efforts. Recently, we and others reported on a high rate of somatic *IDH1-* mutations affecting codon R132 in a large series of brain tumors including oligodendrogliomas, oligoastrocytomas and astrocytomas [13], [14], [15]. Other than LOH1p&19q and *TP53-* mutations, *IDH1-* mutations were found in similar frequencies independent of oligodendroglial or astroglial tumor differentiation and were observed already in low grade tumors. These data seem to suggest, that *IDH1-* mutations may be a tumor initiating event common to both oligodendroglial and astrocytic tumors. *IDH1*- mutations may thus set the scene for genetic and chromosomal instability leading to gene mutations and chromosomal events, which in themselves may influence tumor morphology. To test this hypothesis, we investigated all tumors for *IDH1-* mutations in codon R132.

## Methods

### Ethics Statement

Written informed consent was obtained from each patient according to the research proposals approved by the Institutional Review Boards at Charité University, Berlin, VIVANTES Hospital Neukölln, Berlin and HELIOS Hospital Buch, Berlin, Germany (#187/99, #159/98, #EA2/234/05) at Heidelberg Medical Faculty, Germany (#005/2003), and at the Georg-August-University Göttingen, Germany (#14/9/03) and by the Medical Research Ethics Committee (CCMO) at Radboud University Nijmegen Medical Centre, Netherlands (#2004/127). The present study was approved by the respective ethic committees of all institutions that provided tumor material.

### Tissue Samples and Microdissection

Tumor samples were obtained from a total of 126 patients treated at different institutions between the years 1988 and 2008. DNA from 63 tumors was obtained from the Department of Pathology, Nijmegen Center for Molecular Life Sciences, Radboud University Nijmegen Medical Centre, Nijmegen, Netherlands. 48 tumors were retrieved from Neurosurgery Departments at Charité University Hospital, at VIVANTES Hospital Neukölln and at HELIOS Hospital Buch, Berlin, Germany. 14 tumor specimens were obtained from the Department of Neurosurgery at Ruprecht-Karls Universität, Heidelberg, Germany and 1 tumor was obtained from the Institute of Neuropathology, University Hospital Göttingen, Germany. All tumors were classified and graded according to the guidelines of the World Health Organization [16], [17].

Prior to any DNA extraction particular care was taken to identify tumor tissue based upon morphology and immunohistochemical tissue properties. More so in recurrent tumors in which treatment related reactive tissue reactions sometimes hamper a clear distinction between tumor tissue and treatment related tissue responses. Cases without unequivocal tumor tissue were upfront excluded from DNA extraction and did not undergo any further molecular analysis.

106 gliomas, representing 51 primary tumors with at least one recurrence underwent investigation of their non-microdissected tumor tissue to address the issue of intertumoral clonality during tumor progression. In detail these comprised of 1 pilocytic astrocytoma WHO grade I (PAI), 27 oligodendrogliomas WHO grade II, 25 anaplastic oligodendrogliomas WHO grade III (OIII), 16 oligoastrocytomas WHO grade II (OAII), 9 anaplastic oligoastrocytomas WHO grade III (OAIII), 16 astrocytomas WHO grade II (AII), 3 anaplastic astrocytomas WHO grade III (AIII) and 9 glioblastomas WHO grade IV (GBM). Besides surgical tumor reduction therapeutic regiments included both radiation- and chemotherapy. Sex was equally distributed with 30 female and 32 male patients. At first diagnosis, the youngest patient was 10, the oldest 61. The mean age upon tumor manifestation was 41. Constitutional DNA was available from all patients treated in Berlin and Heidelberg. Leukocyte- and tumor- DNA was extracted using standard methods. 20 of these 126 gliomas were selected for microdissection of predominantly oligodendroglial and astrocytic tumor components based on tumor morphology and immunohistochemical properties. Molecular alterations of these components were investigated separately to address the issue of intratumoral clonality. They included 4 OAII, 6 OAIII, 5 OII and 4 OIII and 1 GBM. 16 tumor specimens were part of 7 tumor pairs with at least one recurrence. The remaining 4 represented single tumors. For microdissection purposes paraffin embedded tumor tissue was used. 16 of 20 selected tumor specimens derived from a total of 11 patients were identified suitable for microdissection. These constituted of primary tumors with at least one recurrent tumor of the same patient or represented primary tumors, only. For microdissection, clearly discernible areas of predominantly astrocytic or oligodendroglial tumor components were identified and marked on H&E slides prior to extraction. In oligodendrogliomas, small areas dominated by tumor cells of astrocytic appearance were microdissected from areas with distinct honeycomb features. 11 consecutive 10 µm sections were used for DNA extraction. After completed microdissection all slides were stained with H&E to exclude extraction of major parts of non- tumorous tissue and to document successful dissection of marked tumor areas. To exclude DNA carry over from one tumor sample to another, DNA was extracted on different occasions. Microsatellite analysis confirmed that leukocyte- and micro- dissected or non- micro- dissected tumor DNA originated from the same patient. All experiments were repeated at least twice to confirm data.

### Analysis of the TP53 Gene

DNA derived from both non- microdissected and microdissected tumor components was analyzed for somatic mutations affecting *TP53*. We analyzed exons 5–8 of *TP53* by SSCP and direct sequencing. Sets of primers spanning exonic and adjacent intronic DNA stretches were employed. Primer sequences and amplification conditions were used as previously described [18]. SSCP analysis was performed on a sequencing apparatus (BlueSeq 400, Boehringer Ingelheim, Germany) using 8%, 10%, 12% and 14% acrylamide gels. Electrophoresis was run at 2–6 W for 15 h. Silver staining of the gels was performed as previously described [19]. Aberrantly migrating SSCP bands were excised, the DNA extracted, purified and re-amplified with the same set of primers. Sequencing was performed on a semiautomatic sequencer (Applied Biosystems, model 377) using the BigDye Terminator Sequencing Kit (Applied Biosystems). Each amplicon was sequenced bi- directionally.

**Table 1:** Non- microdissected cases/data analysis.

tissue	n	Σ(*n*)	LOH1p	%	Σ (*n*)	Σ*%*	LOH19q	%	Σ (*n*)	Σ*%*	LOH1p&19q	%	Σ (*n*)	Σ*%*	*TP53*	%	Σ (*n*)	Σ*%*	*IDH1*	%	Σ (*n*)	Σ*%*
**PA I**	1	***1***	0	0			0	0			0				0				0			
**O II**	27		23	85			24	89			23	85			2	7			24	89		
**O III**	25	52*	20	80	43*	83*	21	84	45*	87*	19	76	42*	81*	5/24	21	7/51*	14*	22	81	46*	88*
**OA II**	16		7	44			8	50			7	44			5	31			10	63		
**OA III**	9	***23*** **∫**	3	33	***10*** **∫**	***43*** **∫**	4	44	***12*** **∫**	***52*** **∫**	3	33	***10*** **∫**	***43*** **∫**	2	22	***7*** **∫**	***30*** **∫**	5	56	***15*** **∫**	***65*** **∫**
**A II**	16		2	13			2	13			1	6			5/14	36			9/12	75		
**A III**	3	***19§***	1	33	***3§***	***16§***	0	0	***2§***	***11§***	0	0	***1§***	***5§***	1	33	***6/17§***	***35§***	2	67	***11/15§***	***73§***
**GBM**	9	***9***	3	33	***3***	***33***	4	44	***4***	***44***	3	33	***3***	***33***	2/5	40	***2/5***	***40***	4/6	67	***4/6***	***67***
**n (total)**	**106**																					

tissue: abbreviation of diagnosis based on histology.

n: number of individual tumors analyzed.

**Σ** (n): sum of cases with respective molecular finding in tumors of similar provenience but different tumor grading (*i.e.* OII/OIII (*), OAII/OAIII (∫), AII/AIII (§)).

**Σ**%: percentage of a molecular finding in summed up tumors of similar provenience but different tumor grading.

### Microsatellite Analysis for LOH 1p&19q

To identify LOH on 1p we used the following tetranuleotide microsatellite markers: D1S1608 (1p36.31), D1S548 (1p36.23), D1S1597 (1p36.21), D1S1592 (1p36.13), and D1S1161 (1p35.1). For determining LOH on 19q, the tetranucleotide markers D19S431 (19q12), D19S433 (19q12), D19S559 (19q13.2), and D19S601 (19q13.33) were implemented. Primer sequences and PCR- conditions correspond to Genome Database entries (www.gdb.org) and were described elsewhere [20]. Amplified DNA was separated on 8% denaturating urea- gels and visualized by silver staining. LOH was scored as previously described [21].

**Table 2:** Microdissected gliomas/data analysis.

tissue	n	differentiation	total	LOH1p	%	Σ (n)	Σ%	LOH19q	%	Σ (n)	Σ%	LOH1p&19q	%	Σ (n)	Σ%	*TP53*	%	Σ (n)	Σ%	*IDH1*	%	Σ (n)	Σ%
**OII**	5	oligodendroglial	5	5	100			5	100			5	100			0	0			4	80		
		*astrocytic*	*5*	*3*	*60*			*3*	*60*			*3*	*40*			*0*	*0*			*4*	*80*		
**OIII**	4	oligodendroglial	4	2	50		78*	2	50		78*	1	25		67*	3	75		33*	2	50		67*
		*astrocytic*	*4*	*1*	*25*	9*	44*	*2*	*50*	9*	56*	*1*	*25*	9*	44*	*3*	*75*	9*	33*	*2*	*50*	9*	67*
**OAII**	4	oligodendroglial	4	1	25			2	50			1	25			2	50			3	75		
		*astrocytic*	*4*	*0*	*0*			*2*	*50*			*0*	*0*			*2*	*50*			*3*	*75*		
**OAIII**	6	oligodendroglial	6	4	67		50∫	4	67		60∫	4	67		50∫	3	50		50∫	5	83		80∫
		*astrocytic*	*6*	*1*	*17*	*10*∫	*10*∫	*3*	*50*	*10*∫	*50*∫	*1*	*17*	*10*∫	*10*∫	*4*	*67*	*10*∫	*60*∫	*5*	*83*	*10*∫	*80*∫
**GBM**	1	oligodendroglial	1	0	0		0	0	0		0	0	0		0	0	0		0	0	0		0
		*astrocytic*	*1*	*0*	*0*	*1*	*0*	*0*	*0*	*1*	*0*	*0*	*0*	*1*	*0*	*0*	*0*	*1*	*0*	*0*	*0*	*1*	*0*
**n (total)**	**20**																						

tissue: abbreviation of diagnosis based on histology.

n: number of individual tumors analyzed.

differentiation: specifies morphology of microdissected tumor components.

**Σ** (n): sum of cases with respective molecular finding in tumors of similar provenience but different tumor grading (*i.e.* OII/OIII (*),OAII/OAIII (∫)); without distinction of oligodendroglial and astrocytic morphology.

**Σ**%: percentage of a molecular finding summed up for therespective tumor components in tumors of similar provenience but different tumor grading (*i.e.* OII/OIII (*), OAII/OAIII (∫)). (*upper field:* frequency in % in the oligodendroglial tumor component;

*lower field*: frequency in % in the astrocytic tumor components.).

### Multiplex Ligation-dependent Probe Amplification (MLPA) for the Detection of Chromosomal Losses on 1p&19q

Loss of chromosomal material on 1p and 19q, equivalent to LOH1p/19q, was detected by MLPA in all tumors obtained from the Netherlands. MLPA was performed as previously described [22].

**Table 3:** Non- microdissected tumors with intertumoral heterogeneity indicated by gain and/or loss of molecular alterations during tumor progression.

Surgery	#	n (P)	P/R	ID	tissue	LOH 1p	LOH 19q	*TP53*	*IDH1*	*IDH1* in detail	*TP53* in detail
NL	1	**1**	**P**	49506	A II	**het**	**het**	n.d.	n.d.	n.d.	n.d.
NL	2	**1**	R	49508	GBM	**pLOH**	**LOH**	n.d.	mut	R132H, G395A	n.d.
NL	3	2	**P**	49528	A II	het	**het**	mut exon 8	mut	R132H, G395A	codon 273, R273C; C817T; **C**GT-> **T**GT
NL	4	2	R1	49526	OA II	het	**LOH**	mut exon 8	mut	R132H, G395A	codon 273; R273C; C817T; **C**GT-> **T**GT
NL	5	2	R2	49576	OA III	het	**LOH**	mut exon 8	mut	R132H, G395A	codon 273, R273C; C817T; **C**GT-> **T**GT
NL	6	**3**	**P**	49532	A II	het	het	**wt**	n.d.	n.d.	wt
NL	7	**3**	R	49534	GBM	het	het	**mut exon 7**	mut	R132C, C394T	codon 242; C242R; T724C; **T**GC-> **C**GC
NL	8	4	**P**	49548	A II	**het**	**het**	n.d.	n.d.	n.d.	n.d.
NL	9	4	R	49550	A II	**pLOH**	**LOH**	wt	wt	wt	wt
HD	10	**5**	**P**	50264	A II	het	**het**	mut exon 5	mut	R132H, G395A	codon 175; C175Y; G527A; T**G**C-> T**A**C
HD	11	**5**	R	50256	GBM	het	**LOH**	mut exon 5	mut	R132H, G395A	codon 175, C175Y; G527A; T**G**C-> T**A**C
HD	12	6	**P**	49200	A II	**het**	pLOH	wt	mut	R132H, G395A	wt
HD	13	6	R	43284	GBM	**LOH**	pLOH	wt	mut	R132H, G395A	wt
B	14	**7**	**P**	24576	O II	**het**	**het**	wt	wt	wt	wt
B	15	**7**	R	25816	O II	**LOH**	**LOH**	wt	wt	wt	wt
B	16	8	**P**	22834	O III	**het**	***pLOH***	mut exon 5	mut	R132H; G395A	codon 135, C135W; TG**C**-> TG**G**
B	17	8	R	24950	O III	**LOH**	***het***	mut exon 5	mut	R132H; G395A	codon 135, C135W; TG**C**-> TG**G**
NL	18	**9**	**P**	49514	A II	**het**	het	***mut exon 5 and exon 8***	mut	R132H, G395A	codon 146; W146Stopp; T**G**G-> T**A**G;codon 281, D281E GA**C**-> GA**G**
NL	19	**9**	R	49516	A III	**pLOH**	het	***mut exon 5***	mut	R132H, G395A	codon 146, W146Stopp; G37A; T**G**G-> T**A**G
NL	20	10	**P**	49560	A II	het	het	wt	***mut***	R132H, G395A	wt
NL	21	10	R	49562	GBM	het	het	wt	***wt***	wt	wt
B	22	**11**	**P**	21818	O II	***LOH***	***LOH***	wt	***mut***	R132H; G395A	wt
B	23	**11**	R	22328	O III	***het***	***het***	wt	***wt***	wt	wt
B	24	12	**R1**	22590	O III	LOH	LOH	mut exon 5	***mut***	R132H; G395A	codon 136, Q136Stopp; C406T; **C**AA -> **T**AA
B	25	12	R2	25172	O III	LOH	LOH	mut exon 5	***wt***	wt	codon 136, Q136Stopp; C406T; **C**AA -> **T**AA
NL	26	**13**	**P**	49366	A II	***LOH***	het	wt	mut	R132H, G395A	wt
NL	27	**13**	R	49552	A II	***het***	het	wt	n.d.	n.d.	wt

Surgery: designates the individual center of tumor operation:

NL: Radboud University Nijmegen Medical Centre, Nijmegen, The Netherlands.

B: Charité Universitätsmedizin, Berlin, Germany.

HD: University Hospital, Heidelberg, Germany.

#: number of all tumors analyzed.

n (P): number of individual tumor pairs.

P/R: identifies primary (P) and recurrent (R) tumor of each tumor pair.

ID: internal tumor ID.

Tissue: abbreviation of diagnosis based on histology.

het.: retained heterozygosity.

LOH: loss of heterozygosity.

pLOH: partial loss of heterozygosity.

mut: mutated *TP53/IDH1.*

wt: wild type *TP53/IDH1.*

n.d.: not determined.

**Bold characters in column:**

n (P): highlight every second tumor pair of individual patients.

P/R: highlight every primary tumor of individual patients.

*TP53* in detail: highlight the position of individual base exchanges in the affected codon of the *TP53*- gene.

In all remaining columns bold characters highlight tumor pairs with novel molecular alterations during tumor progression.

***Bold italic characters:***

Highlight tumor pairs in which molecular alterations of the primary tumor are lost during tumor progression.

### R132 IDH1 Mutation Analysis

The genomic region spanning wild type R132 of *IDH1* was analyzed by direct sequencing, as previously described [15].

## Results

**Figure 1:**
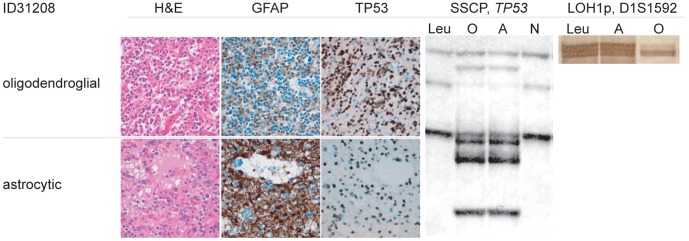
Histology, immunohistochemistry and molecular data of OIII, ID31208. The tumor was significant for a small distinct tumor component of predominantly astrocytic appearance. At time of surgery this astrocytic component was considered too small to allow for the diagnosis of an OAIII. Molecular analysis detected an identical somatic *TP-53*- mutation in both the astrocytic and the oligodendroglial tumor component. However, LOH1p was detected in the oligodendroglial tumor component only. Both oligodendroglial and astrocytic tumor component shared the same *IDH1*- mutation (data not shown) suggesting a common tumor progenitor cell for both components. *Upper panel (left three panels)*: Histology (H&E) and immunohistochemistry (GFAP, TP53) of the predominantly oligodendroglial tumor component. *Lower panel (left three panels)*: Histology (H&E) and immunohistochemistry (GFAP, TP53) of the predominantly astrocytic tumor component. Of note, the oligodendroglial tumor component for the most part lacks GFAP, and nuclear accumulation of TP53- protein is seen in both tumor components. *Middel panel*: SSCP- mutational analysis of the predominantly oligodendroglial (O) and astrocytic (A) tumor component in comparison to patient’s leukocyte DNA (Leu) and a normal control (N). Identical aberrant bands were detected in both tumor components and later confirmed by direct sequencing. For reference see table 4. *Right panel (LOH1p)*: Analysis of polymorphic micro satellite marker D1S1592 revealed retained alleles in the astrocytic tumor component (A). However, the oligodendroglial tumor component (O) was significant for the loss of one allele as compared to the patient’s leukocyte DNA (Leu).

**Figure 2:**
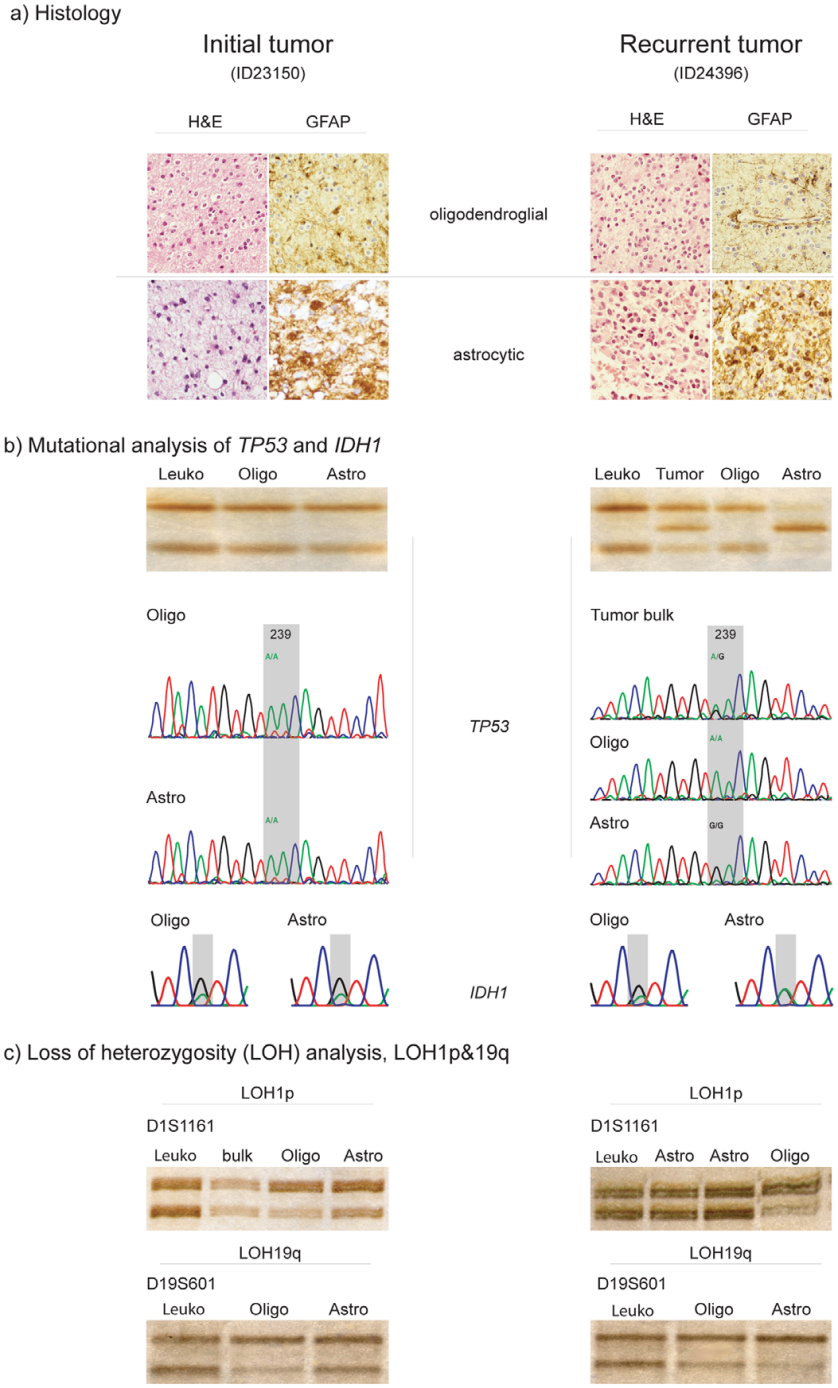
Histology (H&E) and immunohistochemistry (GFAP) (a), SSCP and sequencing (b) and LOH1p/19q analyses (c) of OAII (ID23150, *left panel*, and its recurrence (ID24396), *right panel*. (a) Histology *Upper panel*: H&E and GFAP of predominantly oligodendroglial tumor component in initial tumor, ID23150 *(left)* and its recurrence, ID24396 *(right)*. *Lower panel*: H&E and GFAP of predominantly astrocytic tumor area in initial tumor, ID23150 *(left)* and its recurrence, ID24396 *(right)*. The astrocytic tumor components in initial tumor and recurrence strongly stain positive for GFAP while the respective oligodendroglial tumor areas are almost devoid of GFAP staining. (b) Mutational analysis of *TP53* and *IDH1 Upper panel*: SSCP- analysis of the micro-dissected oligodendroglial and predominantly astrocytic tumor component of the initial tumor, ID23159, *(left)* and non-microdissected and micro-dissected tumor areas in the recurrence, ID24396, *(right)*. No aberrant band was detected in the initial tumor, as compared to the patient’s leukocyte DNA (Leuko). In the recurrent non-microdissected tumor tissue (tumor) and the micro-dissected astrocytic tumor component (Astro) an aberrantly shifted band was detected in exon 7 of the *TP53-* gene. Of note, the aberrant band was confined to the astrocytic tumor component. The oligodendroglial tumor part (Oligo) was significant for wild type *TP53*. *Middle panel*: Direct sequencing of the *TP53*-gene, exon 7 in micro-dissected oligodendroglial (Oligo) and astrocytic (Astro) tumor components in the initial tumor, ID23150 *(left)* and the non-microdissected tissue and the respective micro-dissected areas in the recurrent tumor, ID24396 *(right)*. Sequencing confirms SSCP data for both the initial and the recurrent tumor. While the initial tumor is wild-type- *TP53* in both oligodendroglial and astrocytic tumor component, the non-microdissected tissue of the recurrent tumor reveals heterozygosity for a somatic A->G point mutation in codon239 of the *TP53-* gene. Consequently amino acid aspartate (D) is substituted by asparagine (N). For reference see also table 4. The somatic mutation was confined to the astrocytic tumor component (Astro). The oligodendroglial tumor part remained wild-type- *TP53* (Oligo), suggesting two distinct tumor cell clones. *Lower panel:* Direct sequencing of *IDH1*- gene. Identical G395A mutations in codon 132 (R132H) of IDH1 were detected in both oligodendroglial and astrocytic tumor components. (c) Loss of heterozygosity (LOH) analysis, LOH1p/19q *Upper panel*: LOH1p- analysis of polymorphic microsatellite marker (D1S1161) of the micro-dissected initial tumor, ID23150 *(left)* and its recurrence, ID24396 *(right)*. The initial tumor and its recurrence are significant for LOH1p of the oligodendroglial tumor component only (Oligo). Both alleles were retained in the predominantly astrocytic tumor part (Astro) and the non-microdissected tissue for both the initial *(left)* and the recurrent tumor *(right)*, again indicating two distinct tumor cell populations. *Lower panel*: LOH19q- analysis of polymorphic microsatellite marker (D19S601) of the micro-dissected initial tumor, ID23150 *(left)* and its recurrence, ID24396 *(right)*. Both oligodendroglial (Oligo) and astrocytic (Astro) tumor component of initial and recurrent tumor revealed LOH19q. Patient’s leukocyte DNA (Leuko) was used for reference for both LOH1p- and LOH19q- analyses.

**Figure 3:**
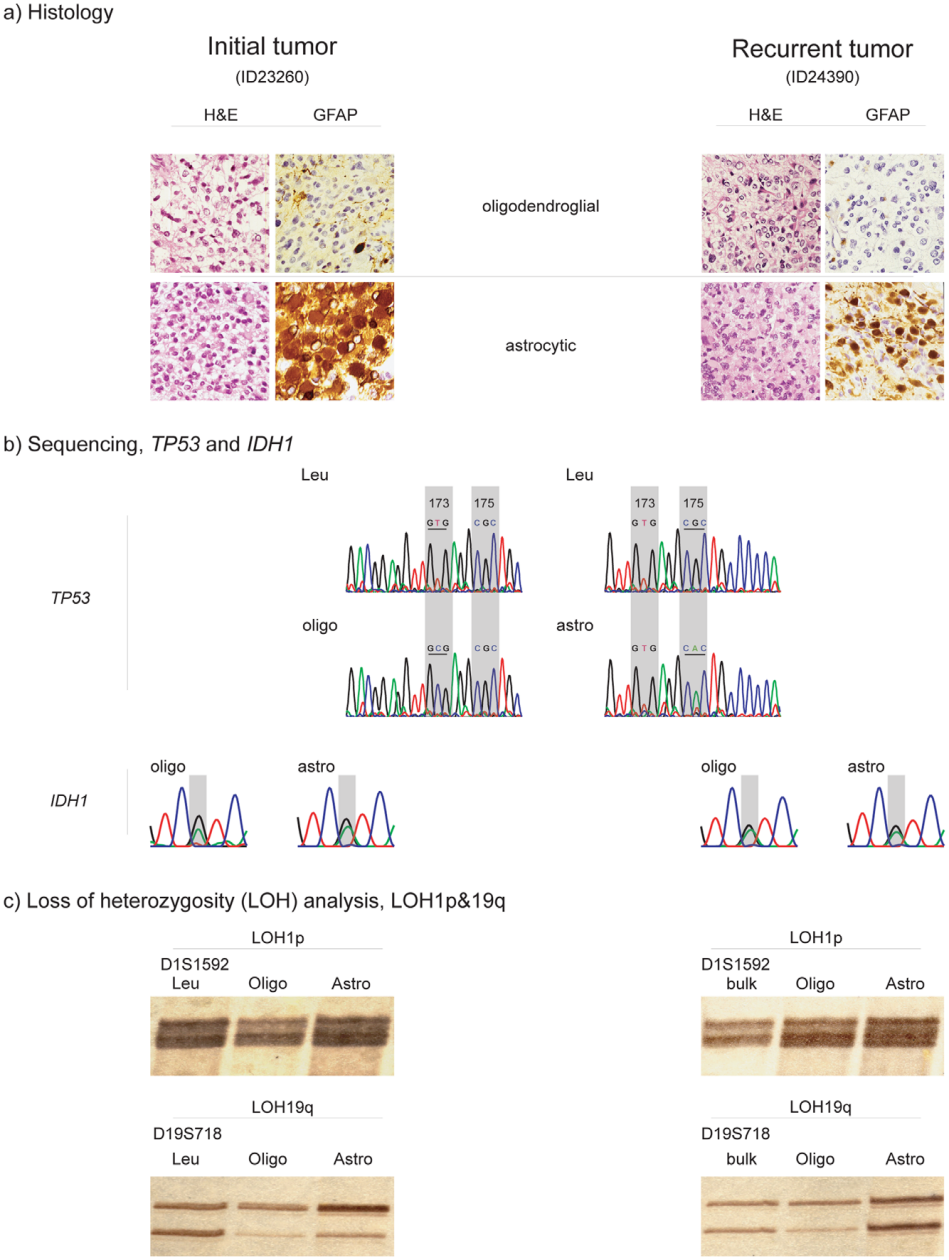
Histology (H&E) and immunohistochemistry (GFAP) (a), sequencing (b) and LOH1p/19q analyses (c) of OAIII (ID23260), *left panel*, and its recurrence (ID24390), *right panel*. (a) Histology *Upper panel*: H&E and GFAP of predominantly oligodendroglial tumor component in initial tumor, ID23260 *(left)* and its recurrence, ID24390 *(right)*. *Lower panel*: H&E and GFAP of predominantly astrocytic tumor area in initial tumor, ID23260 *(left)* and its recurrence, ID24390 *(right)*. The astrocytic tumor components in initial tumor and recurrence strongly stain positive for GFAP while the respective oligodendroglial tumor areas are almost devoid of GFAP- staining. (b) Sequencing, *TP53* and *IDH1 Upper panel*: Direct sequencing of *TP53*- gene, exon 5 of the patient’s leukocyte DNA (Leu) revealing wild- type sequence. *Lower panel:* Direct sequencing of TP53- gene, exon 5 of microdissected intial and recurrent tumor. Of note, the oligodendroglial tumor component harboured a point mutation in codon 173 of the *TP53*- gene and an amino acid substitution of valine -> alanine, while the astrocytic tumor component was significant for a point mutation in codon 175 of the same gene, resulting in an aminoacid substitution arginine -> histidine. Both initial and recurrent tumor harboured the same somatic *TP53*- mutations in the distinct tumor parts, confirming two distinct tumor cell populations in two independent analyses. For reference see also table 4. *Lower panel*: Direct sequencing of *IDH1*- gene. Identical G395A mutations in codon 132 (R132H) of *IDH1* were detected in oligodendroglial and astrocytic tumor components of both the primary tumors and its related recurrence. (c) *Upper panel*: LOH1p- analysis of polymorphic microsatellite marker (D1S1592) of the micro-dissected initial tumor, ID22360 *(left)* and its recurrence, ID24390 *(right)*. The initial tumor and its recurrence are significant for LOH1p of the oligodendroglial tumor component only (Oligo). Both alleles were retained in the predominantly astrocytic tumor part (Astro) in both the initial *(left)* and the recurrent tumor *(right)*, indicating two distinct tumor cell populations. *Lower panel*: LOH19q- analysis of polymorphic microsatellite marker (D19S718) of the micro-dissected initial tumor, ID23260 *(left)* and its recurrence, ID24390 *(right)*. Both oligodendroglial (Oligo) and astrocytic (Astro) tumor component of initial tumor revealed LOH19q. While LOH19q was still detectable in the oligodendroglial tumor component (Oligo) of the recurrent tumor, both alleles were retained in the recurrent astrocytic tumor part, supporting the existence of independent tumor subclones. Patient’s leukocyte DNA (Leuko) was used for reference for both LOH1p- and LOH19q- analyses.

Table S1 summarizes the molecular data relating to all non-microdissected tumors of this study with identical molecular alterations in primary and recurrent tumors. Table 1 depicts the distribution, and frequency of molecular alterations within the individual glioma subtypes in the group of non-microdissected gliomas. Table 2 summarizes the respective data for the microdissected tumors appreciating the findings within the individual tumor components. Table 3 provides detailed information on all molecular alterations of recurrent tumors with evidence of intertumoral heterogeneity, highlighting gains and losses of molecular aberrations during tumor progression. Table 4 provides detailed information of all molecular data relating to the microdissected tumors, highlighting inter- and intratumoral diversities. Figures 1, 2, 3 illustrate data detected in individual tumors highlighting distinct morphological features, immunohistochemical properties and molecular alterations.

**Table 4:** Molecular alterations in recurrent microdissected gliomas.

							oligodendroglial				astrocytic				
	Surgery	#	n (P)	P/R	ID	tissue	LOH1p	LOH19q	*TP53*	*IDH1*	LOH1p	LOH19q	*TP53*	*IDH1*	Figure
**heterogeneous**	B	1	**1**	**P**	23150	OAII	**LOH**	LOH	wt	R132H	**het**	LOH	wt	R132H	2
	B	2	**1**	R1	24396	OAIII	**LOH**	LOH	**wt**	R132H	**het**	LOH	**N239D**	R132H	2
	B	3	2	**P**	22366	OII*	LOH	LOH	wt	R132H	LOH	LOH	wt	R132H	-
	B	4	2	R1	30698	OII	**LOH**	**LOH**	wt	R132H	**het**	**het**	wt	R132H	-
	B	5	2	R2	31848	OII	**LOH**	**LOH**	wt	R132H	**het**	**het**	wt	R132H	-
	B	6	**3**	**R1**	23260	OAIII	**LOH**	LOH	**V173A**	R132H	**het**	LOH	**R175H**	R132H	3
	B	7	**3**	R2	24390	OAIII	**LOH**	**LOH**	**V173A**	R132H	**het**	**het**	**R175H**	R132H	3
	B	8	4	**P**	31208	OIII**	**LOH**	het	R175H	R132H	**het**	het	R175H	R132H	1
**clonal**	B	9	**5**	**P**	21790	OIII	het	*het*	***V173A***	***R132H***	het	*het*	***V173A***	***R132H***	
	B	10	**5**	R	22670	OIII	het	*LOH*	***wt***	***wt***	het	*LOH*	***wt***	***wt***	
	B	11	6	**P**	23772	OAII	het	LOH	wt	wt	het	LOH	wt	wt	
	B	12	**7**	**P**	22588	OAIII	LOH	LOH	wt	R132H	LOH	LOH	wt	R132H	
	B	13	8	**P**	31162	OII	LOH	LOH	wt	R132H	LOH	LOH	wt	R132H	
	B	14	**9**	**P**	28182	OII	LOH	LOH	*wt*	wt	LOH	LOH	***wt***	wt	
	B	15	**9**	R	28906	OIII	LOH	LOH	*R258Q*	wt	LOH	LOH	***R258Q***	wt	
	B	16	10	**P**	30166	OAIII	het	het	wt	wt	het	het	wt	wt	
	B	17	10	R	31850	GBM	het	het	wt	wt	het	het	wt	wt	
	B	18	**11**	P	22622	OAII	het	het	A161T	R132H	het	het	A161T	R132H	
	B	19	**11**	R1	22824	OAII	het	het	A161T	R132H	het	het	A161T	R132H	
	B	20	**11**	R2	23798	OAIII	het	het	A161T	R132H	het	het	A161T	R132H	

* Tumor not suitable for microdissection.

** Oligodendroglioma with discernible tumor component of predominantly astrocytic morphology.

Surgery: designates the individual center of tumor operation:

B: Charité Universitätsmedizin, Berlin, Germany.

#: number of all tumors analyzed.

n (P): number of individual tumor pairs.

P/R: identifies primary (P) and recurrent (R) tumor of each tumor pair.

ID: internal tumor ID.

Tissue: abbreviation of diagnosis based on histology.

het.: retained heterozygosity.

LOH: loss of heterozygosity.

wt: wild type *TP53/IDH1.*

Figure: Figure illustrating respective molecular data.

**Bold characters:**

n (P): highlight every second tumor pair of individual patients.

P/R: highlight every primary tumor of individual patients.

In all remaining columns bold characters highlight tumor pairs with heterogeneous distinct molecular alterations in their respective astrocytic and oligodendroglial tumor component.

*Italic characters:*

Highlight tumor pairs with novel molecular alterations during tumor progression.

***Bold italic characters:***

Highlight tumor pairs in which molecular alterations of the primary tumor are lost during tumor progression.

### Frequency and Intertumoral Dynamics of TP53 Mutations in Non-microdissected Tissues

The pure oligodendrogliomas of this study (OII n = 27; OIII n = 25) harbored somatic *TP53-* mutations in 14% (7/51). 2 mutations were detected in OII (7%) and 5 in OIII (21%), respectively. The oligoastrocytomas (OAII n = 16; OAIII n = 9) revealed *TP53-* mutations in 30% (7/23). 5 mutations were detected in OAII (31%) and 2 in OAIII (22%). The astrocytomas (AII n = 16; AIII n = 3) harbored *TP53* mutations in 35% (6/17). 5 mutations were found in AII (36%) and 1 mutation was detected in one of the 3 AIII (33%). *TP53-* mutations were detected in 2/5 GBM (40%). The single PAI proofed wild-type for *TP53*. Clonality for the *TP53-* status could be established for all but two tumor pairs (96%; n = 51). In one case, upon tumor progression from an AII (ID49532) to a secondary GBM (ID49534) a novel *TP53-* mutation affecting exon 7 was detected. In another case, an AII (ID49514) recurred as an AIII (ID49516). In the recurrent tumor a somatic mutation affecting exon 8, present in the primary tumor, was lost. Of note, a second *TP53-* mutation affecting exon 5 remained present in both the primary and the recurrent tumor of this case. For reference see Tables 1 and 3.

### Frequency and Intra- and Intertumoral Dynamics of TP53- mutations in Microdissected Gliomas

None of all OII with discernible and microdissectable tumor components of oligodendroglial and astrocytic morphology harbored a *TP53-* mutation (0%, n = 5). 75% (n = 3/4) of the OIII harbored *TP53* mutations in both microdissected tumor components, respectively. Taken together, in the predominantly oligodendroglial differentiated tumors *TP53-* mutations were evenly distributed between the tumor components reminiscent of oligodendroglioma and astrocytoma (n = 3/9; 33%).

In the small group of OAII two somatic alterations of *TP53* were detected in the oligodendroglial and astrocytic tumor component, each (n = 2/4; 50%). The OAIII revealed three *TP53-* mutations in their oligodendroglial (50%; n = 3/6) and four mutations (67%; n = 4/6) in their astrocytic tumor components. Thus, OAII and OAIII together harbored *TP53-* mutations in 50% (n = 5/10) of their oligodendroglial tumor component, whereas the astrocytic tumor component revealed *TP53-* mutations in 60% (n = 6/10). No *TP53-* mutation was detected in the single GBM analyzed here. Intratumoral clonality for *TP53*, represented by identical molecular alterations affecting both oligodendroglial and astrocytic tumor components, could be established in 60% (n = 12/20) of all microdissected tumors. Divergent *TP53* status was established in a total of three tumors (15%).

OAII (ID23150) was *TP53* wild-type. Its recurrent anaplastic tumor OAIII (ID24396), however, revealed a *TP53-* mutation confined to the astrocytic tumor component (Figure 2).

The oligodendroglial and astrocytic tumor components in two recurrent tumors of an OAIII (ID23260 and ID24390) revealed divergent *TP53*- mutations. While V173A was detected in the microdissected oligodendroglial tumor components of both tumors, R175H was present in both astrocytic tumor components of the same tumors (Figure 3).

Progression associated divertive intertumoral alterations for *TP53* were detected in two tumor pairs. Besides the observation of a progression associated novel *TP53-* mutation in the astrocytic tumor component of OAIII, ID24396, (Figure 2), the V173A mutation found in both microdissected components of primary OIII, ID21790, was no longer detectable upon recurrence (OIII, ID22670). For reference see also Tables 2 and 4.

### Frequency and Intertumoral Dynamics of Isolated and Combined LOH1p/19q in Non-microdissected Tissues

LOH1p was detected in 83% (43/52) of all oligodendrogliomas. In detail, 85% of OII (23/27) and 80% of OIII (20/25) revealed LOH1p. LOH1p was combined with LOH19q in all but one case, ID22834. Thus, for the oligodendrogliomas, the frequency of combined LOH1p/19q was 81% (42/52). Additional isolated LOH19q was seen in one OII (4%, n = 1/27) and in two OIII (8%, n = 2/25). In OAII and OAIII the frequency of LOH1p was 43% (10/23). As LOH1p was combined with LOH19q in all these cases, combined LOH1p/19q had the same frequency as LOH1p alone (43%, 10/23). 44% of OAII (7/16) and 33% of OAIII (3/9) revealed LOH1p and LOH19q, respectively. Additional isolated LOH19q was observed in one OAII (6%, n = 1/16) and one OAIII (11%, n = 1/9). 16% of AII and AIII (3/19) revealed LOH1p, with 13% of AII (2/16) and 33% of AIII (1/3), respectively. Combined LOH1p/19q was confined to one AII (6%, 1/16). Combined LOH1p/19q was detected in 33% of GBM (3/9). All of these were secondary GBM preceded by an AII in two cases (ID49508, ID43284) and by an OAIII in one case (ID49520). Isolated LOH19q was seen in one additional GBM (11%, n = 1/9). The single PAI of this study showed no evidence for LOH1p or LOH19q.

Data on LOH1p/19q was available for all 106 non- microdissected recurrent gliomas representing 51 tumor pairs of primary tumor with at least one recurrent tumor. LOH1p/19q status remained unchanged upon tumor recurrence in 80% (41/51) of the tumor pairs comprising of a total of 85 tumors (80%, n = 85/106). Divergent results between primary tumor and its recurrences were observed in 10 tumor pairs (20%, n = 10/51) represented by 21 tumors (20%, n = 21/106). Additional isolated LOH1p or LOH19q confined to the recurrent tumor detected in three (30%, n = 3/10) and two (20%, n = 2/10) of the glioma pairs, respectively. Additional combined LOH1p/19q confined to the recurrent tumor was observed in another three (30%, n = 3/10) of the glioma pairs. In three tumor pairs (30%, n = 2/10) an isolated LOH1p, an isolated LOH19q and a combined LOH1p/19q were no longer detectable in the recurrent tumors (ID24950, ID22328, ID49552). Interestingly an isolated partial LOH19q of OIII, ID22834 was no longer detectable upon tumor recurrence, whereas isolated LOH1p was confined to the recurrence (ID24950). Of note, despite these intertumoral differences affecting LOH1p and LOH19q both primary (ID22834) and recurrent tumor (ID24950) harbored an identical *TP53*- and *IDH1*- mutation. For reference see Table 1 and 3.

### Frequency and Intra- and Intertumoral Dynamics of Isolated and Combined LOH1p/19q in Microdissected Gliomas

The tumor components dominated by oligodendroglial morphology in OII all revealed combined LOH1p/19q (100%, n = 5/5). The microdissected tumor components reminiscent of an astrocytoma in two of these tumors revealed retained alleles for both 1p and 19q (40%, n = 2/5). In OIII combined LOH1p/19q of both the microdissected oligodendroglial and astrocytic tumor components was observed in 25% (n = 1/4). Isolated LOH1p was observed in one additional oligodendroglial tumor component of an OIII (ID31208, Figure 1). Isolated LOH19q was found in both the oligodendroglial and the predominantly astrocytic tumor component of one recurrent OIII (ID22670). The total frequency of isolated LOH1p and LOH19q was 78% for the respective oligodendroglial tumor components of all oligodendrogliomas (n = 7/9). For the tumor components with more astrocytic morphology the frequency for isolated LOH1p was 44% (n = 4/9) and for isolated LOH19q 56% (n = 5/9). Combined LOH1p/19q was observed in 67% (n = 6/9) of the oligodendroglial and in 44% (n = 4/9) astrocytic tumor components. Intratumoral divergent results relating to LOH1p and/or LOH19q were observed in the two recurrent tumors of OII, ID22366 and the OIII, ID31208. The two recurrent tumors of OII, ID22366, consistently revealed combined LOH1p/19q in their oligodendroglial and retained alleles for 1p and 19q in their tumor components with predominantly astrocytic morphology (ID30698, ID31848). The primary tumor itself suggested LOH1p/19q in both tumor components, however clear- cut microdissection in this particular tumor was hindered by a more diffuse growth pattern of the two tumor components, so that carry over from oligodendroglial tumor components to the astrocytic tumor components could not be fully excluded. In OIII, ID31208, the isolated LOH1p was confined to the tumor component with typical oligodendroglial morphology (Figure 1). Intertumoral changes during tumor progression relating to the LOH19q- status were observed in the recurrent OIII, ID22670. While both alleles on 19q were retained in the primary tumor OIII, ID21790, the recurrent tumor (ID22670) was significant for isolated LOH19q (Table 4). Combined LOH1p/19q was observed in 25% (1/4) of the oligodendroglial tumor components in OAII and in none of their tumor components with astrocytic morphology. In OAIII combined loss of LOH1p/19q was detected in 67% (4/6) in the oligodendroglial and in 17% (1/6) of the astrocytic tumor components. The total frequency of combined LOH1p/19q in all OA was 50% (5/10) for the oligodendroglial and 10% (1/10) for the astrocytic tumor components. Isolated LOH19q was detected in the oligodendroglial tumor component of one OAII and the astrocytic tumor components of two OAII and 2 OAIII, respectively. Intratumoral heterogeneity affecting the LOH1p- and/or LOH19q- status was observed in 40% (4/10) of the oligoastrocytic tumors. While the oligodendroglial tumor components of OAII, ID23150 and its recurrent tumor OAIII, ID24396 showed combined LOH1p/19q, the respective astrocytic tumor components revealed retained alleles on 1p and isolated LOH19q (Figure 2). A similar observation was documented for OAIII, ID23260 and its recurrence OAIII, 24390 (Figure 3). The oligodendroglial tumor components again revealed combined LOH1p/19q in both the primary and the recurrent tumor, whereas the astrocytic tumor component retained both alleles on 1p and revealed isolated LOH19q in the primary and retained alleles on 19q in the recurrent tumor. The single secondary GBM, ID31850 retained both maternal and paternal alleles on both 1p and 19q. For reference see Table 2 and 4.

### Frequency and Intertumoral Dynamics of R132 IDH1- mutation in Non-microdissected Tissues

Somatic mutations affecting codon 132 of *IDH1* were detected in 88% (n = 46/52) of all oligodendrogliomas. In detail, OII harbored *IDH1*- mutations in 89% (n = 24/27) and OIII in 81% (n = 22/25). *IDH1*- mutations were detected in 63% of OAII (n = 10/16) and in 56% in OAIII (n = 5/9), respectively. In all OAII and OAIII harbored *IDH1*- mutations in 65% (n = 15/23). 75% (n = 9/12) of AII and 67% (n = 2/3) of AIII revealed *IDH1-* mutations. The frequency of *IDH1* mutations in all AII and AIII together was 73% (n = 11/15). *IDH1-* mutations were detected in 67% (n = 4/6) GBM. All mutations affected secondary glioblastomas. The single PAI of this study proofed wild-type for codon 132 of *IDH1*. *IDH1*- mutation data was available from 99 of the 106 non-microdissected tumors, representing a total of 47 tumor pairs with at least one recurrent tumor. Of these, 92 tumors (95%) representing 44 tumor pairs (94%) revealed an identical *IDH1*- status in their primary and recurrent tumors. The *IDH1*- mutational status changed in the recurrent tumor of three tumor pairs (6%). In all three instances the *IDH1*- mutation observed in the primary tumor was no longer detected in the respective recurrent tumors (ID49562, ID22328 and ID25172). For reference see Tables 1 and 3.

### Frequency and Inter- and Intertumoral Dynamics of R132 IDH1- Mutation in Microdissected Gliomas

OII and OIII harbored *IDH1*- mutations in 67% (n = 6/9). In detail, *IDH1*– mutations affected 80% (n = 4/5) of OII and 50% (n = 2/4) OIII. The group of OAII and OAIII revealed *IDH1*- mutations in a frequency of 80% (n = 8/10) with mutations in 75% (n = 3/4) of OAII and 83% (n = 5/6) of OAIII, respectively. The single GBM proofed wild- type for codon 132 of *IDH1*. Comparison of *IDH1*- mutational data was possible for a total of 7 tumor pairs with at least one recurrent tumor comprising of 16 tumors. The *IDH1*- mutational status remained unchanged in all but one tumor pair (86%) represented by 14 tumors in total (87%). In one tumor pair the *IDH1*- mutation present in the primary tumor was no longer detectable upon tumor recurrence (ID22670). Of note, when present *IDH1*- mutations always affected both, the oligodendroglial and the astrocytic tumor component of the microdissected tumors.

### Combined Data Analysis of Non-microdissected and Microdissected Gliomas Regarding TP53- mutation, LOH1p/19q and R132 Mutational Status of IDH1

Intertumoral differences affecting *TP53,* LOH1p/19q, and *IDH1* in combination was seen in two of the 13 non- microdissected tumor pairs with intertumoral heterogeneity (Table 3). Partial LOH1p was detected associated with tumor recurrence in AIII, ID49516). While the primary lesion AII, ID49514 revealed retained alleles for both 1p and 19q and two somatic mutations affecting exon 5 and exon 8 of the *TP53-* gene, upon tumor recurrence the *TP53-* mutation affecting exon 8 was no longer present in the tissue of the recurrent AIII, ID49516.

Of note, both primary and recurrent tumor shared identical mutations affecting exon 5 of *TP53* and the R132H mutation in *IDH1*. The persistence of both the *TP53-* mutation affecting exon 5 and the *IDH1*- mutation together with the introduction of a partial LOH1p in the recurrent tumor strongly argue for the analysis of tumor tissue in both the analyses of the primary and the recurrent tumor. In the second tumor pair (OII, ID21818 and OIII, ID22328) the molecular alterations, *i.e.* LOH1p/19q and *IDH1*- mutation observed in the primary lesion (ID21818) were no longer detectable in the recurrence (ID22328). Even though prior to DNA isolation it was made sure that all tissues contained tumor, in this particular case, it can not be completely ruled out that the tissue morphology of the recurrence mimicked tumor tissue due to reactive changes following combined radio- chemotherapy. Intertumoral heterogeneity upon tumor recurrence affecting more than one of the analyzed molecular parameters was seen in one of seven microdissected tumor pairs (Table 4). Isolated LOH19q was detected in the recurrent tumor, OIII, ID22670 whereas its primary lesion, OIII, ID21790 revealed both alleles on 19q. In the same recurrent tumor both the *TP53*- mutation and the *IDH1*- mutation present in the primary lesion were no longer detectable. All these alterations affected both microdissected tumor components of this tumor. A combination of inter- and intratumoral differences affecting more than one molecular parameter was detected in two of the seven microdissected tumor pairs (Table 4). Upon tumor progression OAIII, ID24396 revealed a novel *TP53*- mutation confined to the tumor component with predominant astrocytic morphology. While the microdissected tumor components with oligodendroglial morphology of both, the primary, OAII, ID23150 and the recurrent tumor, OAIII, ID24396, revealed a combined LOH1p/19q for all investigated informative markers, the astrocytic tumor component retained both alleles for 1p and showed an isolated LOH19q (Figure 2). Recurrent glioma pair OAIII, ID23260 and OAIII ID24390 revealed combined LOH1p/19q, a *TP53*- mutation affecting codon 173 (V173A) and R132H *IDH1-* mutation in their respective microdissected oligodendroglial tumor component. The astrocytic tumor component of both these tumors was significant for retained alleles on 1p, a *TP53*- mutation affecting a different codon of the *TP53* gene (R175H) and an identical R132H *IDH1*- mutation. While the astrocytic tumor component of OAIII, ID23260 revealed LOH19q, retained alleles on 19q were documented for the recurrent lesion OAIII, ID24390. The persistence of the distinct *TP53*- mutational status affecting codon 173 and codon 175, respectively, in both the oligodendroglial and astrocytic tumor components of OAIII, ID23260 and OAIII, ID24390 excludes gross cross- contamination of these tumor components during analyses (Figure 3).

Of note, both microdissected tumor pairs with detected inter- and intratumoral heterogeneities harbored the most frequent R132H *IDH1*- mutation in both their respective oligodendroglial and astrocytic tumor component, suggesting *IDH1-* mutation to be a common tumor initiating event in oligodendrogliomas, astrocytomas and oligoastrocytomas.

## Discussion

Molecular testing increasingly influences therapeutic decisions also in neuro- oncology. Testing usually is confined to tissue won during the first operational procedure. Therapeutic decisions upon tumor recurrence usually rely on these findings postulating molecular stability within the recurrent tumor. The combined analysis of *TP53-,* LOH1p/19q and *IDH1* in a large series of recurrent gliomas aimed at elucidating the type and frequency of inter- and intratumoral dynamics of these molecular alterations during tumor progression. It also addressed a potential impact that therapeutic strategies may have on the molecular phenotype of the recurrent tumor, and *vice versa*, that an altered molecular phenotype during tumor progression may have on therapeutic efforts in treating the recurrent tumor. Microdissection of a small series of recurrent gliomas additionally investigated the coexistence of distinct subpopulations of tumor cell clones within gliomas with distinct morphologies (*i.e.* astrocytic vs. oligodendroglial). The majority of matched primary and recurrent tumors revealed clonal stability. However, clinical tumor progression was accompanied by an altered molecular phenotype in a number of recurrent cases. These could be divided into either the emergence of novel molecular alterations associated with tumor progression or the loss of previously detected alterations due to an overgrowth by a different tumor cell clone. Thus, novel *TP53*- mutations were documented in three recurrent gliomas. Previously not detected isolated LOH1p or LOH19q were found in three recurrent tumor pairs, respectively, and combined LOH1p/19q confined to the recurrent glioma was documented in another three recurrent gliomas.

Of note, while oligodendroglial differentiation and morphology is strongly tied to combined 1p/19q deletion in primary glioma lesions, loss at either or both loci, particularly in the context of more complex copy number changes during tumor progression may simply reflect progressing malignancy. Thus, a primary tumor with astrocytic tumor morphology that develops 1p/19q loss over time (as in case 49550) does not necessarily evolve into a fundamentally oligodendroglial neoplasm, but rather remains an astrocytic neoplasm that features additional molecular alterations that may reflect progressing malignancy.

The fact that novel molecular alterations that impact therapeutic strategies due to their association with chemosensitivity and a favorable outcome, *i.e.* combined LOH1p/19q, do develop in a small subset of recurrent gliomas argues for molecular work-up of recurrent tumors whenever possible. Therapeutic management may benefit by directly targeting the altered molecular features of the recurrent tumor. Of note, *IDH1-* mutations, if present, occurred already in low grade tumors and none of the recurrent tumors revealed newly developed *IDH1*- mutations. This underscores the notion that *IDH1*- mutations are a decisive factor for tumor initiation [23], [24]. The occurrence of additional molecular events during tumor progression also fits our understanding of a step-wise tumor development in gliomas [25].

However, the loss of previously detected molecular alterations affecting *TP53* (n = 2), *IDH1* (n = 4), isolated LOH1p (n = 1), isolated LOH19q (n = 2) and combined LOH1p/19q (n = 1) was also observed at tumor recurrence. Interestingly, one primary tumor with combined LOH1p/19q (ID21818) revealed retained alleles for 1p and 19q upon recurrence, ID22328, following cytoreductive surgery and chemotherapy, suggesting that chemosensitive tumor subclones may disappear during targeted chemotherapy. Intratumoral selection pressure and tumor cell microenvironment may be responsible for the outgrowth of one tumor cell clone over another, accompanied by the disappearance of a previously detectable tumor cell clone. As an example, during early passages of cultured tumor cells, the mutant p53 content increases with passaging due to outgrowth of mutant clones from a heterogeneous starting population [26]. In addition, *de novo* p53 mutations appeared during culture [26]. These experiments show that the microenvironment is crucial for the selection of the dominant tumor cell clone. It is conceivable that glioma therapy, *i.e.* chemotherapy and/or radiotherapy, also influences the pre-existing microenvironment and that an altered microenvironment favors a different tumor cell clone leading to the outgrowth of one and the disappearance of another. Thus the disappearance of LOH1p/19q in recurrent glioma, ID24576, may be due to the susceptibility of the tumor cell clone with LOH1p/19q to chemotherapy and the outgrowth of a more chemotherapy resistant tumor cell population.

A similar phenomenon has recently been described for the acquired resistance to epidermal growth factor receptor tyrosine kinase inhibitors in non-small-cell lung cancers [27]. Sensitive detection methods have identified a proportion of tyrosin- kinase- naive tumors that carry T790M, a resistant tumor cell clones that may be selected during the exposure to gefitinib or erlotinib. Also, gefitinib- sensitive breast cancer has been found to acquire resistance through novel point mutations in *HER2/neu* [28]. What is more, therapy- induced disappearance of previous molecular alterations in the same tumor has also been described before, *i.e.* the loss of epidermal growth factor receptor gene mutation in lung cancer with natural resistance to gefitinib (IRESSA) [29]. In principle, the non-detection of previously established molecular features may root in the microdissection technique itself. However, the simultaneous persistence of previously detected molecular features, documented in the primary tumor successfully identified the recurrent tissue also as tumor. While inter- tumoral heterogeneity thus reflects either the emergence of novel molecular events during tumor progression or may be due to environmental changes within the tumor triggered by therapeutic measures, evidence of intratumoral heterogeneity may shed some light onto the biology driving tumor initiation and morphology. To address this issue we looked for intratumoral heterogeneity in microdissected recurrent mixed gliomas. In an effort to increase the likelihood to detect diverting molecular features of distinct tumor areas with either oligodendroglial or astrocytic morphology we selected LOH1p/19q- and *TP53*- mutational analyses, as was done in similar previous studies [30], [31]. In the past, clonality analyses on gliomas have been performed repeatedly with ambiguous results. The majority of these studies concluded clonal origin by investigating either morphologically distinct tumor components [18], [32], [33] or by comparing surgical with autopsy material [33] from the same patient. The coexistence of two independent tumor initiating cell populations was postulated in a case of synchronous malignant gliomas [34]. Clonality and proteomic analyses highlighted an independent origin of a pleomorphic xanthoastrocytoma with anaplastic features and an anaplastic oligoastrocytoma in the same patient [34]. Gliomatosis cerebri is a neuroepithelial neoplasm with extensive infiltration of the brain [16]. As gliomatosis cerebri by definition affects multiple lobes of the brain clonality analyses in tissue of gliomatosis patients allows the analysis tumor areas as distant from each other as is possible excluding cross contamination due to insufficient microdissection technique. The same is true in rare cases of metastatic satellites of primary brain tumor [18]. In gliomatosis patients data is limited to a few reports in which monoclonal origin dominated [33], [35], [36], [37]. A case with spinal metastasis of a glioblastoma was also demonstrated to be monoclonal [18]. However, regional polyclonality within single gliomatosis cerebri cases has been reported [33], [37].

**Figure 4:**
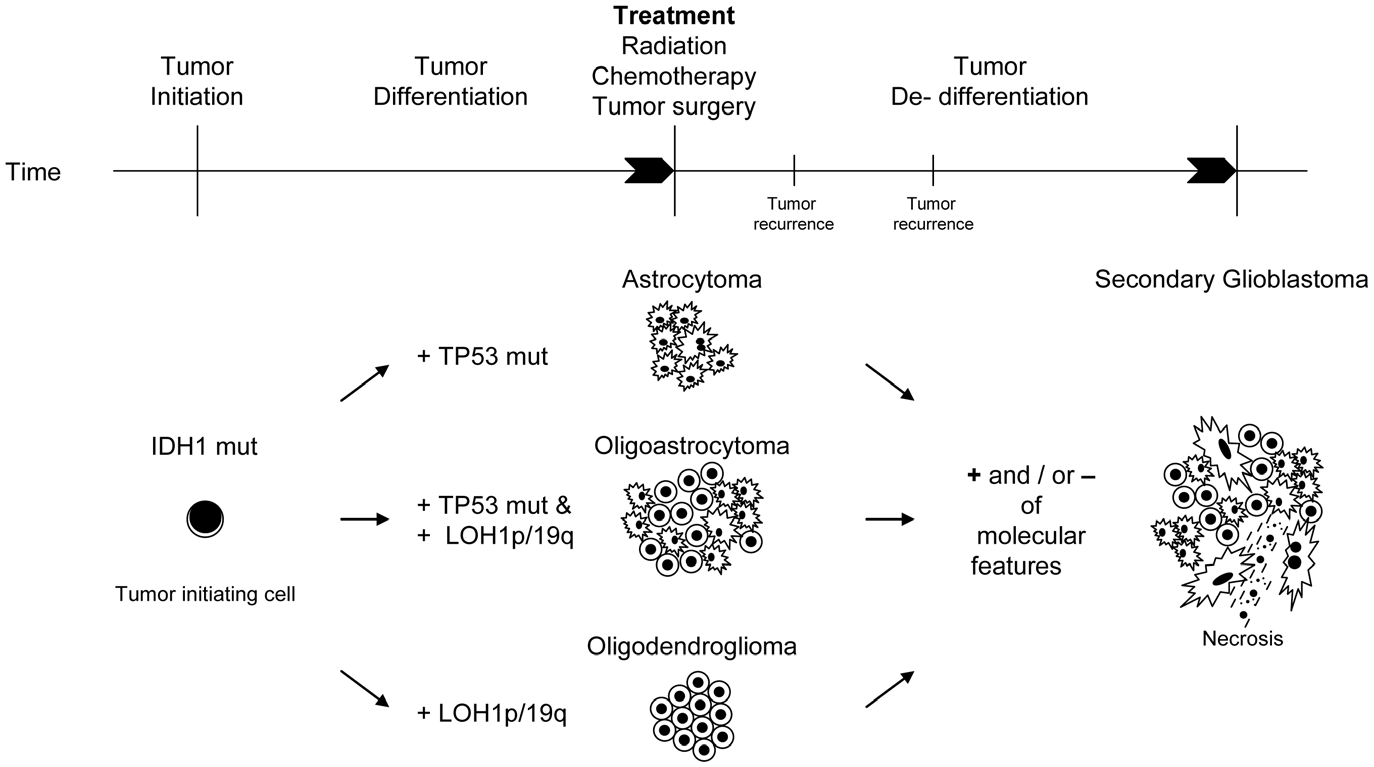
Potential hierarchy of molecular events during tumor initiation, differentiation and de-differentiation upon tumor recurrence in pure and mixed gliomas. Time line of tumor development from left to right. Tumor initiation occurs by the introduction of an *IDH1*- mutation in a common tumor progenitor cell. Tumor differentiation is significant for astrocytoma- typical molecular alterations in pure astrocytomas (*i.e. TP53*- mutations) and oligodendroglioma- typical alterations in pure oligodendrogliomas (*i.e.* LOH1p&19q). Of note, in pure oligoastrocytomas, *TP53*- mutations and LOH1p&19q may occur separately in the tumor parts with the respective morphology (oligodendroglial vs. astrocytic). Following tumor therapy with surgery, radio- and chemotherapy additional molecular events may occur at tumor recurrence. Also, previous molecular alterations might disappear due to overgrowth of therapy- resistant tumor clones or the disappearance of tumor- susceptible tumor cell clones. Tumor dedifferentiation finally leads to the morphological picture of a secondary glioblastoma with or without an oligodendroglial component as the common morphological endstage of malignant gliomas.

As for mixed gliomas intratumoral clonal diversity was reported from clonality studies in a recurrent oligoastrocytoma with extended recurrence-free interval [38]. Also, small series of oligoastrocytomas have been investigated for their clonality status [30], [31]. Our results confirm previous observations that the oligoastrocytomas are predominantly of monoclonal origin [30], [31]. Also in keeping with our own data a small subset of tumors with divergent molecular features has been detected in these studies [30], [31]. This has been explained in these studies by distinct tumor progenitor cells for the oligodendroglial and the astrocytic tumor component, respectively [30], [31]. However, none of these studies included *IDH1* mutation analysis. While *TP53*- mutations and LOH1p/19q are frequent and distinct alterations for astrocytic and oligodendroglial tumor components, respectively, *IDH1*- mutations were found in similar frequency in both [15], suggesting a similar function of IDH1 during the tumorigenesis of these tumors. Also, *IDH1*- mutations were detected in high frequencies in low grade gliomas of astrocytic and oligodendroglial differentiation identifying *IDH1*- mutations as a common early and important event during tumor development [15]. This has led to the proposal of a common tumor progenitor cell for both oligodendrogliomas and astrocytomas, both harboring *IDH1*- mutations [23]. It has further been suggested that the advent of *TP53-* mutations in these tumor progenitor cells ensues astrocytic morphology while LOH1p/19q is more closely associated with oligodendroglial appearance [23]. So far, the molecular steps leading to the development of oligoastrocytomas has not yet been appreciated in that model. Our series of microdissected and non-microdissected gliomas confirms both the high frequency and the early time point of *IDH1*- mutations in glioma development. Interestingly, those microdissected tumors, which were significant for divergent and distinct molecular alterations in their oligodendroglial and astrocytic components in relation to *TP53*- mutations and LOH1p/19q, shared the same *IDH1*- mutations not only in their primary tumor but also in the respective recurrences. This implies a common tumor progenitor cell population carrying the *IDH1*- mutation which later is separated by the occurrence of a *TP53*- mutation for the astrocytic and a combined LOH1p/19q for the oligodendroglial tumor component, similar to the model for tumor development in astrocytomas and oligodendrogliomas. If altered IDH1- enzyme activity or the novel enzymatic product 2-hydroxyglutarate [39] due to the mutation directly set the stage for an increased chromosomal and genetic instability of affected gliomas facilitating additional molecular events remains to be elucidated. As not all low- grade gliomas featured *IDH1*- mutations, we presume that tumor- initiating events other than IDH1 alterations exist. The results of our investigation provide a conceivable line of molecular events (see Figure 4). *IDH1*- mutations or other molecular events with similar properties mark tumor initiation and provide a microenvironment with increased chromosomal and genetic instability allowing additional alterations to occur. These additional alterations may not only convey growth- advantages over other tumor cell clones but may also in part influence tumor morphology itself, explaining the observation of LOH1p/19q in oligodendroglial and a *TP53*- mutation in astrocytic tumor components of one tumor. Tumor de-differentiation during tumor progression, clonal expansion and overgrowth, influenced by changes in the microenvironment of the tumor during therapeutic efforts may explain the disappearance of some and the prevalence of other tumor cell populations. While necessary for tumor initiation the importance of mutated *IDH1* for tumor maintenance remains to be elucidated. We observed IDH1 wild- type reversion in four cases. In two of these cases alterations for 1p/19q and mutated *TP53* prevailed, identifying the tissue as tumor tissue. However, even though special care was taken to ensure tumor tissue for DNA extraction, it cannot be completely excluded that the *IDH1*- mutations in the recurrent tumors merely escaped detection. A limited number of tumor cells within the sample used for DNA extraction or the co- presence of contaminating normal cells (*i.e.* lymphocytes, brain tissue, endothelial cells) are two feasible reasons that could explain our results. As all four cases harbored the most frequent R132H mutation in their primary lesions, that has recently become detectable by immunohistochemistry [40], it would have been intriguing to implement this antibody, as it succeeds in the detection single mutated cells within tumor tissue or the tumor edge infiltrating adjacent normal brain tissue [41]. However, for a large number of cases in this retrospective study all tumor tissue that had been available was used to allow for the molecular analyses of this or other ongoing studies. Unfortunately, these four cases were among them. Thus, future studies are warranted to confirm or disprove our observation of wild type reversion of *IDH1* in recurrent gliomas that harbored *IDH1*- mutations in their primary tumor, to fully understand its importance for tumor maintenance and during tumor progression.

### Conclusions

We conclude that recurrent gliomas are predominantly of monoclonal origin. We confirm that *IDH1*- mutations seem to initiate gliomagenesis. Our data suggest that true oligoastrocytomas share the same progenitor cell population carrying an identical *IDH1*- mutation with a small subset of tumors with *TP53-* mutations in the astrocytic and combined LOH1p/19q in the oligendroglial tumor component, only accessible for identification by microdissection. And finally the existence of divertive subclones in gliomas *per se* and evidence of treatment- driven molecular alterations in recurrent gliomas may directly impact future therapeutic decisions for the individual patient. We therefore advocate tissue extraction whenever possible also from recurrent gliomas to allow for molecular testing and ensure a patient- and tumor- tailored therapy.

## Supporting Information

Table S1 **Summary of experimental data relating to all non- microdissected tumors with identical molecular alterations in primary and recurrent tumor.** Surgery: designates the individual center of tumor operation; NL: Radboud University Nijmegen Medical Centre, Nijmegen, The Netherlands; B: Charité Universitätsmedizin, Berlin, Germany; HD: University Hospital, Heidelberg, Germany; #: number of all tumors analyzed; n (P): number of individual tumor pairs; P/R: identifies primary (P) and recurrent (R) tumor of each tumor pair; ID: internal tumor ID; tissue: abbreviation of diagnosis based on histology; het.: retained heterozygosity; LOH: loss of heterozygosity; pLOH: partial loss of heterozygosity; mut: mutated *TP53/IDH1;* wt: wild type *TP53/IDH1*; n.d.: not determined*; IDH1* in detail: base exchange and codon position of *IDH1-* mutation*; TP53* in detail: base exchange and codon position of *TP53*- mutation*;* SSCP: single strand conformation polymorphism analysis.
(XLS)
